# Simultaneous Determination of 15 Sulfonate Ester Impurities in Phentolamine Mesylate, Amlodipine Besylate, and Tosufloxacin Tosylate by LC-APCI-MS/MS

**DOI:** 10.1155/2019/4059765

**Published:** 2019-10-07

**Authors:** Bo Jin, Kaijing Guo, Tingting Zhang, Tong Li, Chen Ma

**Affiliations:** Institute of Material Medica, Chinese Academy of Medical Sciences and Peking Union Medical College, Beijing 100050, China

## Abstract

Sulfonate esters have been recognized as potential genotoxic impurities (PGIs) in pharmaceuticals. An LC-MS/MS method was developed and validated for the simultaneous determination of 15 sulfonate esters, including methyl, ethyl, propyl, isopropyl, and *n*-butyl esters of methanesulfonate, benzenesulfonate, and *p*-toluenesulfonate in drug products. The method utilized atmospheric pressure chemical ionization (APCI) in multiple reaction monitoring (MRM) mode for the quantitation of impurities. The method employed an ODS column as the stationary phase and water-acetonitrile as the solvents for gradient elution without derivatization steps. The method was specific, linear, accurate, precise, and robust. Recoveries of the sulfonic esters from three drug matrices were observed in the range of 91.6∼109.0% with an RSD of not greater than 17.9% at the concentration of the LOQ and in the range of 90.4%∼105.2% with an RSD of not greater than 7.1% at the concentration of 50 ng/mL for the methanesulfonates and 10 ng/mL for the benzenesulfonates and *p*-toluenesulfonates. The LOD was not greater than 15 ng/mL, 2 ng/mL, and 1 ng/mL for the methanesulfonate, benzenesulfonate, and *p*-toluenesulfonate esters, respectively. This method was sufficiently sensitive to detect the 15 PGIs in the phentolamine mesylate tablet, amlodipine besylate tablet, and tosufloxacin tosylate tablet. This analytical method is a direct, specific, rapid, and accurate quality control tool for the determination of the 15 sulfonate esters that are most likely to exist in drug products.

## 1. Introduction

Sulfonate esters, which may reside in active pharmaceutical ingredients (APIs), originate from unexpected reactions between the sulfonic acids used as salt-forming counterions and short-chain alcoholic agents employed in the same synthetic process. Genotoxicity of different sulfonate esters, including methanesulfonate, benzenesulfonate, and *p*-toluenesulfonate, has been suggested by computer-assisted structural considerations and in vitro approaches involving the Ames mutagenicity test and the micronucleus test [1]. Because of the actual or possible carcinogenicity and mutagenicity of potential genotoxic impurities (PGIs) to humans, a threshold of toxicological concern (TTC) of 1.5 *μ*g/day was suggested by the International Council for Harmonisation of Technical Requirements for Pharmaceuticals for Human Use (ICH) M7 (R1) for an individual genotoxic impurity over a long duration of treatment (>10 years) [2]. In addition, the control strategy of PGIs is based on the understanding of the product and process and utilization of risk management principles [2], which requires the pharmaceutical industry to implement process controls and demands analytical chemists to develop methods capable of monitoring the fate of PGIs during chemical synthesis [3].

The low-acceptable intake level of PGIs in drug substances/products sets higher demands and challenges for establishing analytical methods with respect to sensitivity and specificity. Considering the differentiated volatility of sulfonate esters, gas chromatography- (GC-) based and liquid chromatography- (LC-) based methods have been developed. Methyl, ethyl, and isopropyl mesylate esters in APIs or final products have mostly been quantified by gas chromatography-mass spectrometry (GC-MS) [4–6]. A high-performance liquid chromatography-ultraviolet (HPLC-UV) method with derivatization has been utilized to determine methyl and ethyl methanesulfonates in methanesulfonic acid with a LOQ of 0.6 ppm [7]. HPLC with single-stage MS (HPLC-MS) or tandem mass spectrometry (HPLC-MS/MS) has also demonstrated its application in methanesulfonates [8], benzenesulfonates, and *p*-toluenesulfonates [9]. However, these methods only focused on one or two classes of sulfonate esters, and a more generic or platform-based approach [10] is practically needed for the screening of potentially existing sulfonate impurities during process control. A good attempt of this approach was an in-situ derivatization-headspace-GC/MS method established for the simultaneous determination of methyl, ethyl, and isopropyl esters of methanesulfonate, benzenesulfonate, *p*-toluenesulfonate, and other sulfates in APIs [11]. In addition, an indirect derivatization method using hydrophilic interaction chromatography-electrospray ionization mass spectrometry (HILIC-ESI-MS) was conducted to detect 16 of the most encountered alkyl sulfonates and dialkyl sulfates in APIs [12]. Moreover, Guo et al. [13] demonstrated LC hyphenated with tandem mass spectrometry methods to quantify 12 sulfonate esters. They developed two HPLC methods to separately determine aliphatic sulfonate esters and aromatic sulfonate esters.

The aim of the current study was to develop one simple and rapid separation method for analysis of multiple sulfonate esters, which does not require derivatization. An HPLC-APCI-MS/MS method was developed for the simultaneous determination of 15 PGIs, including the methyl, ethyl, propyl, isopropyl, and butyl esters of methanesulfonates, benzenesulfonates, and *p*-toluenesulfonates in pharmaceuticals. The method was rapid, specific, accurate, and sensitive for the detection of mesylate, besylate, and tosylate drug products.

## 2. Materials and Methods

### 2.1. Materials

Reference standards were obtained as follows: the methyl (99%) and ethyl (98%) esters of methanesulfonate, methyl ester of benzenesulfonate (98%), and ethyl (98.0%) and isopropyl (97%) esters of *p*-toluenesulfonate were purchased from Sigma-Aldrich Co. (St. Louis, MO, USA); the propyl (98.0%) and isopropyl (98.0%) esters of methanesulfonate, ethyl (98.0%) and n-butyl (98.0%) esters of benzenesulfonate, and methyl (98.0%), propyl (98.0%), and n-butyl (97.0%) esters of *p*-toluenesulfonate were obtained from Tokyo Chemical Industry Co., Ltd. (Chuo-ku, Tokyo, Japan); the propyl ester of benzenesulfonate (97%) was provided by Bide Pharmatech Ltd. (Shanghai, China); the isopropyl ester of benzenesulfonate (98%) was purchased from Adamas Reagent Co., Ltd. (Shanghai, China); and the *n*-butyl ester of methanesulfonate (98.5%) was provided from J&K Scientific Ltd. (Beijing, China).

HPLC grade methanol, acetonitrile, phosphoric acid (85%), and ammonium acetate (NH_4_Ac) were purchased from Fisher Scientific Products (Fair Lawn, NJ, USA). HPLC grade acetic acid (HAc) was obtained from Tedia company, Inc. (Fairfield, OH, USA). Ultrapure water was obtained from a Milli-Q water purification system (Millipore, Bedford, MA, USA).

Three drug samples were obtained from pharmacies. The product name, manufacturer, batch number, strength, recommended upper limit daily dose, and limit of PGIs based on 1.5 *μ*g/day, and the maximum daily dosage are listed in Table 1.

### 2.2. Instrumentation

Chromatographic separations were performed on a Shimadzu UFLC-20AD XR system with an autoinjector and binary solvent manager (Shimadzu, Tokyo, Japan). The column was a Kromasil C18 column (250 mm × 4.6 mm, 5 *μ*m, AkzoNobel, Bohus, Sweden), and the oven temperature was set to 30°C. The gradient elution was employed with solution A (water) and solution B (acetonitrile). The gradient solvent program was set as follows: time/% solution B, 0/40, 5.0/90, 7.5/90, 8.0/40, and 11.0/40. The flow rate was set to 1.0 mL/min, and the injection volume was 10 *μ*L.

The Shimadzu UFLC-20AD XR system was coupled online to an Applied Biosystems Sciex Qtrap 5500 mass spectrometer (MDS-Sciex, Concord, Canada) with an atmospheric pressure chemical ionization (APCI) interface. The typical operating source conditions for the MS scan in negative APCI mode were optimized as follows: corona current, −5 *μ*A; vaporizer temperature, 300°C; curtain gas (nitrogen), 20 psi; nebulizer pressure, 40 psi; collision exit potential (CXP), −8 V; entrance potential (EP), 10 V; declustering potential (DP), −10 V; and collision energy (CE) of 25 eV for methanesulfonate esters and 30 eV for benzenesulfonate and *p*-toluenesulfonate esters. The MRM ion pairs were *m/z* 95 ⟶ 80, *m/z* 157 ⟶ 93, and *m/z* 171 ⟶ 107 for methanesulfonate, benzenesulfonate, and *p*-toluenesulfonate esters, respectively. The HPLC fraction corresponding to the API that eluted before 3.3 min was diverted to waste. Analyst 1.6 software was used for the control of equipment, data acquisition, and analysis.

### 2.3. Standards and Test Solutions

A standard stock solution (10 mg/mL) of each of the 15 sulfonate esters was prepared by dissolving the accurately weighed reference substance in methanol and storing at 2°C–6°C until further use. A series of mixed standard working solutions were freshly prepared from the standard stock solution and diluted with methanol containing 0.25% HAc before use, giving concentrations in the range of LOQ-200 ng/mL for propyl methanesulfonate, LOQ-150 ng/mL for other methanesulfonate esters, LOQ-400 ng/mL for benzenesulfonate esters, and LOQ-100 ng/mL for *p*-toluenesulfonate esters. The limit of detection (LOD) and limit of quantitation (LOQ) were estimated at a signal-to-noise ratio of 3 : 1 and 10 : 1, respectively. Precision was determined by analyzing six replicates at the concentrations of the LOQ and 50 ng/mL. In the recovery test, the reference samples of the standards were added at concentrations of LOQ and 50 ng/mL for the methanesulfonate esters and 10 ng/mL for the benzenesulfonate and *p*-toluenesulfonate esters in powdered tablets with six replicates. The matrix effect was quantitated by comparing the peak area of six replicates of the mixed standards (100 ng/mL) added into an extracted drug matrix with that of the standards at the same concentration in the absence of matrix. Both the reference samples from the recovery test and the extracted matrices from the matrix effect evaluation followed the sample preparation procedure described in Section 2.4. The stability of the sample solutions containing the 15 analyte standards presenting in the three extracted drug matrices as well as in the solvents were prepared and kept at room temperature in the dark. The stability of the sample solutions was measured by recording the peak area of every analyte at hourly intervals for 24 h.

### 2.4. Sample Preparation

A quantity of powdered drug tablets equivalent to 10 mg of the drug substance was weighed accurately and extracted with 5 mL of methanol containing 0.25% HAc in an ultrasound bath for 10 min. The extracted solution was filtered through a 0.45 *μ*m membrane syringe filter (ANPEL Laboratory Technologies Inc., Shanghai, China), and the subsequent filtrate was used as the test solution.

## 3. Results and Discussion

### 3.1. Sample Solvent Selection

Stability of the sample solution is an important consideration in a solvent selection. We selected acetonitrile, acetonitrile with acetic acid, methanol, and methanol with acetic acid as sample solutions, and the 15 analytes were stable for less than 4 h in acetonitrile with or without acetic acid, for 16 h in methanol, and for 24 h in methanol with 0.25% acetic acid. As shown in Table 2, the ratios of the peak areas of the 15 analytes in methanol with 0.25% acetic acid after 24 h to those at 0 h in different matrices were in the range of 0.92∼1.15. Stable times of these sample solutions were longer than those reported in the literature [8, 13], making the current method more practicable. Kakadiya et al. [8] reported that methyl and ethyl methanesulfonate in a solution of acetonitrile and water (70 : 30, v/v) can remain stable for up to 9 h in the presence of lopinavir and for 11 h in the presence of ritonavir. The methanesulfonate esters in the presence of imatinib mesylate in acetonitrile/15 mM ammonium acetate at pH 3.4 (20 : 80, v/v) and *p*-toluenesulfonate esters in the presence of vinpocetine in acetonitrile/water (80 : 20, v/v) were found to be stable for an hour [13].

### 3.2. Method Development

**Figure 1:**
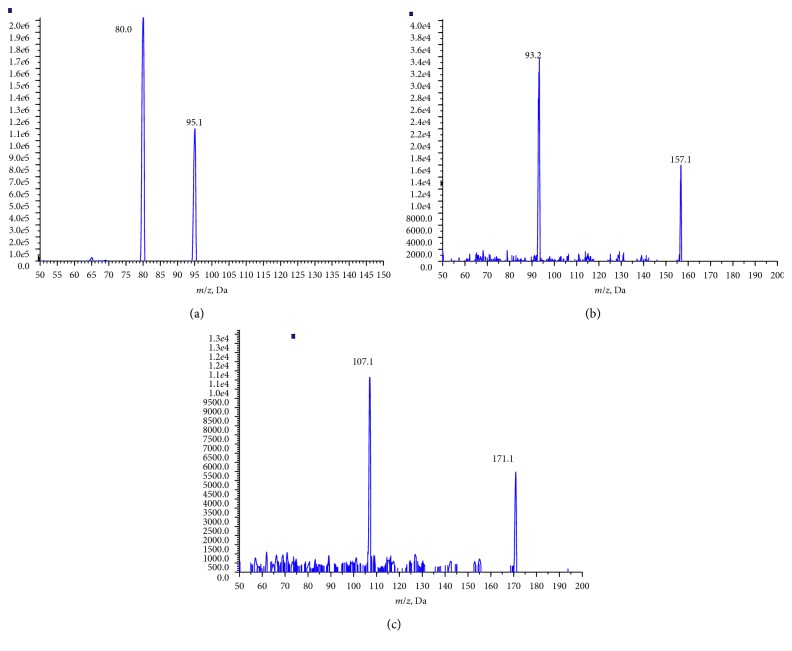
(a) An MS/MS spectrum of a methanesulfonate ester. (b) An MS/MS spectrum of a benzenesulfonate ester. (c) An MS/MS spectrum of a *p*-toluenesulfonate ester.

A series of MS parameters were optimized to solutions of each standard to compare MRM ion responses in the mass spectra. The precursor and product ions showed poor sensitivity and reproducibility in ESI ion mode. APCI was shown to be a stable and sensitive ionization technique for the rapid determination of PGIs. APCI was performed in negative mode. Corona current varied from −3 *μ*A to −5 *μ*A and −5 *μ*A produced the strongest signal response. The vaporizer temperature had a significant effect on the ion response. Temperatures of 200∼500°C were carefully evaluated and 300°C was optimal. In addition, a declustering potential set at 10 V was suitable for all the 15 analytes. The optimal collision energy was found to be 25 eV for methanesulfonate esters and 30 eV for benzenesulfonate and *p*-toluenesulfonate esters. The esters in each group shared the same multiple reaction monitoring (MRM) ion pairs (Figure 1). All the studied methanesulfonate esters shared [M-alkyl]^−^ ions as precursor molecular ions and [M-alkyl-CH_3_]^−^ as dominant daughter ions, so *m/z* 95 ⟶ *m/z* 80 was selected as the MRM ion pair for aliphatic sulfonate esters. Aromatic sulfonate esters exhibited [M-alkyl]^−^ ions and [M-alkyl-SO_2_]^−^ ions as MRM ion pairs, i.e., *m/z* 157 ⟶ *m/z* 93 for benzenesulfonate esters and *m/z* 171 ⟶ *m/z* 107 for *p*-toluenesulfonate esters. Because of the same MRM ion pairs, the esters in each group need to be separated by chromatography before MS analysis.

**Figure 2:**
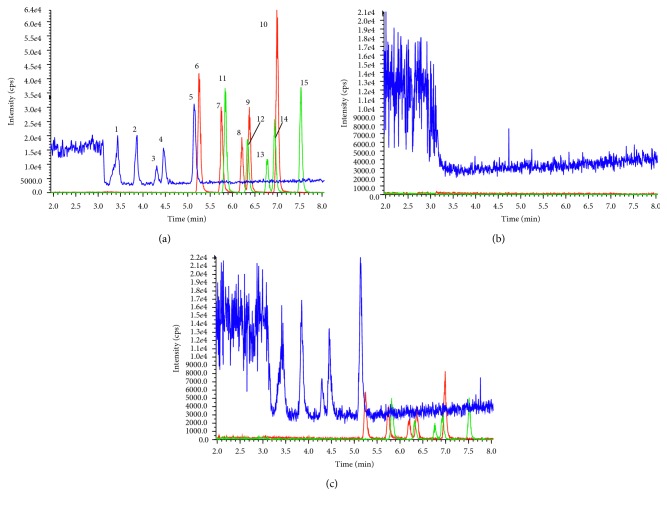
(a) The 15 analytes in methanol containing 0.25% HAc (80 ng/mL): peaks 1–5, methyl, ethyl, propyl, isopropyl, and butyl methanesulfonate, respectively; peaks 6–10, methyl, ethyl, propyl, isopropyl, and butyl benzenesulfonate, respectively; and peaks 11–15, methyl, ethyl, propyl, isopropyl, and butyl *p*-toluenesulfonate, respectively. (b) An MRM chromatogram of the extracted drug product of phentolamine mesylate. (c) An MRM chromatogram of the 15 analytes (50 ng/mL methanesulfonate esters and 10 ng/mL benzenesulfonate and *p*-toluenesulfonate esters) presented in the phentolamine mesylate tablet.

Octadecyl silica (ODS) columns in different sizes, including 75 mm × 3.0 mm (2.2 *μ*m), 100 mm × 3.0 mm (2.7 *μ*m), and 250 mm × 4.6 mm (5 *μ*m), were employed and compared, and the column with a 250 mm length showed a better separation capability to resolve the 15 sulfonate esters within a short period of time. The investigation of mobile phase included water, 0.1% HAc, and NH_4_Ac-HAc buffer in varying combinations with methanol or acetonitrile. The results showed that the MS responses of the standards were higher in acetonitrile than in methanol, and there was no difference between acetonitrile with water, 0.1% HAc, or acetate buffer, so the combination of acetonitrile and water was selected as the mobile phase. Furthermore, different gradient programs as well as the column temperature were optimized to finally obtain a satisfactory separation of the 15 analytes (Figure 2).

### 3.3. Method Validation

#### 3.3.1. Selectivity and Chromatography

The validated assay was evaluated by a solvent blank, and the extracted analyte-free drug products showed no significant interference with the 15 sulfonate analytes. Typical MRM chromatograms of the sulfonate esters in the solvent and in the presence of an extracted analyte-free drug matrix are shown in Figure 2.

#### 3.3.2. Sensitivity and Linearity

The LOD, LOQ, linear range, and equation are summarized in Table 3. Propyl methanesulfonate and benzenesulfonate showed lower sensitivity than the other alkyl esters in the same group. The LOD was not greater than 15 ng/mL, 2 ng/mL, and 1 ng/mL for the methanesulfonate, benzenesulfonate, and *p*-toluenesulfonate esters, respectively. All correlation coefficients were greater than 0.99.

#### 3.3.3. Accuracy and Precision

The recoveries of mixed standard solutions at the LOQ and 50 ng/mL methanesulfonate esters or 10 ng/mL benzenesulfonate and *p*-toluenesulfonate esters presented in the three tablets are summarized in Table 4. The recoveries of the sulfonic esters from the three drug matrices were observed in the range of 91.6∼109.0% with an RSD of not greater than 17.9% at the concentration of the LOQ and in the range of 90.4%∼105.2% with an RSD of not greater than 7.1% at the concentration of 50 ng/mL for the methanesulfonates and 10 ng/mL for the benzenesulfonates and *p*-toluenesulfonates. The precisions of the 15 sulfonate esters at the LOQ and 50 ng/mL were all below 6.9% (Table 2).

#### 3.3.4. Matrix Effect

No significant matrix effect was observed in the three tablets for all the analytes. The matrix effect of the 15 sulfonate esters in the three extracted matrices were all observed in the range of 86.4%∼111.1%, with an RSD of less than 10.0%.

#### 3.3.5. Ruggedness

Ruggedness of the method was evaluated by adjusting the flow rate, column temperature, initial proportion of mobile phase B (acetonitrile), vaporizer temperature, and collision energy. The results found that the 15 sulfonate esters were well separated when the parameters were in the following ranges: flow rate within 0.9∼1.0 mL/min, column temperature within 30∼40°C, initial proportion of mobile phase B in the gradient elution within 38%∼42%, vaporizer temperature within 290∼310°C, and collision energy within 23∼27 eV for methanesulfonate esters and within 28∼32 eV for benzenesulfonate and *p*-toluenesulfonate esters.

### 3.4. Application to Drug Products

The results showed that the 15 sulfonate esters were not detected in the phentolamine mesylate tablet, amlodipine besylate tablet, and tosufloxacin tosylate tablet. The LOD values in this method were not greater than 7.5 *μ*g/g, 1 *μ*g/g, and 0.5 *μ*g/g for the methanesulfonate, benzenesulfonate, and *p*-toluenesulfonate esters, respectively, which corresponded to the drug products. As the ICH M7 [2] suggested, the TTC of the drug products was 1.5 *μ*g/day for a long duration of treatment. The upper limit daily dose was 60 mg, 10 mg, and 0.6 g for phentolamine mesylate, amlodipine, and tosufloxacin tosylate, respectively (Table 1). This method was sufficiently sensitive to quantify the 15 genotoxic impurities in the tested sulfonate drugs.

## 4. Conclusions

A simple, rapid, and reliable HPLC-APCI-MS/MS method has been developed for the determination of three types of sulfonate esters in pharmaceuticals. The advantage of the proposed method was that it required no derivatization and was capable of simultaneously separating the 15 sulfonic acid esters most likely to exist, covering methanesulfonates, benzenesulfonates, and *p*-toluenesulfonates within a single HPLC run. This method was sufficiently sensitive to detect the 15 PGIs in the phentolamine mesylate tablet, amlodipine besylate tablet, and tosufloxacin tosylate tablet and can be further studied for its application to other drug substances or products.

## Figures and Tables

**Table 1:** Drug products tested.

Product	Manufacture	Batch number	Strength	Recommended upper limit daily dosage	Limit of PGIs based on 1.5 *μ*g/day
Amlodipine besylate tablet	Pfizer Inc. (NY, NY, USA)	20170520	5 mg	10 mg	150 *μ*g/g
Phentolamine mesylate tablet	Beijing Shuguang Pharmaceutical Industrial Co., Ltd. (Beijing, China)	170215	40 mg	60 mg	25 *μ*g/g
Tosufloxacin tosylate tablet	Chifeng Wanze Pharmaceutical Co., Ltd. (Chifeng, Neimenggu, China)	170524	0.15 g	0.6 g	2.5 *μ*g/g

**Table 2:** Stability of sample solutions after 24 h in different matrices and precisions of the 15 sulfonate esters at two concentration levels (*n* = 6).

	Sample solution stability				Precision	
	A_24 h_/A_0 h_ in different matrices				Precision at different levels (RSD, %)	
	Methanol with 0.25% HAc	Phentolamine mesylate tablet	Amlodipine besylate tablet	Tosufloxacin tosylate tablet	LOQ	50 ng/mL
Methyl methanesulfonate	1.07	1.06	1.14	1.15	6.2	5.6
Ethyl methanesulfonate	1.03	1.13	1.03	1.01	4.8	3.7
Propyl methanesulfonate	1.07	1.04	1.08	0.99	4.0	4.0
Isopropyl methanesulfonate	1.17	1.15	0.99	1.14	4.1	5.4
Butyl methanesulfonate	1.20	1.05	1.05	1.01	5.7	4.0
Methyl benzenesulfonate	1.09	1.08	1.11	1.05	5.6	2.3
Ethyl benzenesulfonate	1.12	1.09	1.19	1.04	6.5	3.8
Propyl benzenesulfonate	1.04	0.92	0.95	0.94	6.6	4.5
Isopropyl benzenesulfonate	1.09	1.03	1.09	0.97	4.9	3.5
Butyl benzenesulfonate	1.14	1.07	1.03	0.98	6.0	3.9
Methyl *p*-toluenesulfonate	1.07	1.10	1.00	1.01	5.3	1.2
Ethyl *p*-toluenesulfonate	1.15	1.04	1.03	1.04	6.0	4.0
Propyl *p*-toluenesulfonate	1.04	1.00	1.12	1.01	6.9	4.3
Isopropyl *p*-toluenesulfonate	1.12	0.98	1.06	1.05	4.4	1.3
Butyl *p*-toluenesulfonate	1.15	1.14	1.03	0.94	5.8	3.9

**Table 3:** LOD, LOQ, linear range, and regression equations for the 15 sulfonate analytes.

Analyte	LOD (ng/mL)	LOQ (ng/mL)	Linear range (ng/mL)	Regression equation with correlation coefficient (*r*)
Methyl methanesulfonate	10	15	15–150	*y* = 1083.9*x* + 11041 (*r* = 0.9987)
Ethyl methanesulfonate	10	15	15–150	*y* = 811.42*x* + 3743.2 (*r* = 0.9991)
Propyl methanesulfonate	15	40	40–200	*y* = 279.07*x* + 4104.8 (*r* = 0.9958)
Isopropyl methanesulfonate	10	15	15–150	*y* = 646.27*x* + 5004.1 (*r* = 0.9976)
Butyl methanesulfonate	10	12	12–150	*y* = 1330.4*x* + 2849.7 (*r* = 0.9985)
Methyl benzenesulfonate	1	2	2–400	*y* = 2067.7*x* − 208.46 (*r* = 0.9996)
Ethyl benzenesulfonate	1	2	2–400	*y* = 1414.6*x* − 203.62 (*r* = 0.9997)
Propyl benzenesulfonate	2	5	5–400	*y* = 865.35*x* + 547.21 (*r* = 0.9998)
Isopropyl benzenesulfonate	1.5	3	3–400	*y* = 1367.5*x* + 454.06 (*r* = 0.9998)
Butyl benzenesulfonate	1	1.5	1.5–400	*y* = 2849.6*x* − 706.01 (*r* = 0.9997)
Methyl *p*-toluenesulfonate	0.5	1.5	1.5–100	*y* = 1719.3*x* + 505.87 (*r* = 0.9995)
Ethyl *p*-toluenesulfonate	1	2	2–100	*y* = 881.38*x* − 234.18 (*r* = 0.9999)
Propyl *p*-toluenesulfonate	1	2	2–100	*y* = 546.8*x* + 219.39 (*r* = 0.9995)
Isopropyl *p*-toluenesulfonate	1	2	2–100	*y* = 1144*x* − 467.86 (*r* = 0.9995)
Butyl *p*-toluenesulfonate	0.5	1.5	1.5–100	*y* = 1646.6*x* + 74.082 (*r* = 0.9998)

**Table 4:** Recoveries and RSDs of the 15 sulfonate esters in the three tablets (%, *n* = 6).

	Phentolamine mesylate tablet				Amlodipine besylate tablet				Tosufloxacin tosylate tablet			
	LOQ	RSD	C^a^	RSD	LOQ	RSD	C^a^	RSD	LOQ	RSD	C^a^	RSD
Methyl methanesulfonate	104.9	7.8	97.3	4.5	98.0	12.3	102.3	5.2	100.6	12.6	101.6	6.0
Ethyl methanesulfonate	93.9	10.8	99.8	5.2	102.8	13.8	101.3	6.2	101.1	4.5	104.2	4.5
Propyl methanesulfonate	101.9	12.1	99.2	3.6	101.5	11.9	102.9	6.8	100.5	7.6	94.0	7.1
Isopropyl methanesulfonate	109.0	10.5	99.5	5.3	102.4	10.8	100.4	4.6	98.0	10.8	103.5	3.8
Butyl methanesulfonate	103.4	13.9	104.2	3.9	99.8	7.0	102.0	5.8	101.0	13.5	99.9	3.9
Methyl benzenesulfonate	99.8	8.9	99.9	2.1	101.7	11.8	103.5	3.0	100.9	5.0	99.8	3.3
Ethyl benzenesulfonate	101.5	9.7	96.3	5.0	99.3	12.4	101.4	5.9	98.8	13.6	99.4	3.0
Propyl benzenesulfonate	98.8	9.5	103.2	3.1	98.9	13.3	95.1	5.8	96.9	13.1	105.1	2.8
Isopropyl benzenesulfonate	99.8	6.6	99.3	3.6	100.2	11.6	105.1	6.1	99.9	5.9	104.6	2.9
Butyl benzenesulfonate	96.9	8.2	101.4	2.1	97.1	8.6	92.7	4.9	95.8	5.2	95.3	4.2
Methyl *p*-toluenesulfonate	103.3	9.2	99.0	1.4	98.2	6.3	100.4	4.7	100.9	3.7	102.8	3.5
Ethyl *p*-toluenesulfonate	105.4	15.0	103.1	2.4	100.6	4.4	100.1	4.4	106.2	13.9	102.8	3.1
Propyl *p*-toluenesulfonate	100.3	3.1	101.1	6.6	99.8	9.6	92.0	3.8	100.4	17.9	94.6	2.4
Isopropyl *p*-toluenesulfonate	96.6	8.4	99.1	2.3	91.6	9.5	90.4	6.6	96.9	14.1	97.1	1.9
Butyl *p*-toluenesulfonate	102.0	5.6	99.8	2.0	99.9	11.3	97.5	1.4	100.5	12.2	105.2	1.9

^a^C: 50 ng/mL methanesulfonate esters and 10 ng/mL benzenesulfonate and *p*-toluenesulfonate esters.

## Data Availability

All data included in this study are available upon request from the corresponding author.
